# Developing a code of practice for literature searching in health sciences: a project description

**DOI:** 10.29173/jchla29409

**Published:** 2022-04-01

**Authors:** Brooke Ballantyne Scott, Susan Baer, Ashley Farrell, Pat Lee, Jackie MacDonald, Danielle Rabb, Marcus Vaska

**Affiliations:** 1 Library Services, Fraser Health, Westminster, BC.; 2 Library Services & Knowledge Management, Saskatchewan Health Authority, Regina, SK.; 3 Information Specialist at University Health Network, Toronto, ON.; 4 Nova Scotia Health Library, Halifax, NS.; 5 IM/IT, South Shore Health, Bridgewater, NS; 6 Research Information Services, CADTH, Ottawa, ON.; 7 Knowledge Resource Service, Alberta Health Services, Calgary, AB.

## Abstract

**Introduction:**

Libraries have provided mediated search services for more than forty years without a practice standard to guide the execution of searches, training of searchers, or evaluation of search performance. A pan-Canadian group of librarians completed a study of the literature on mediated search practices from 2014-2017 as a first step in addressing this deficit.

**Methods:**

We used a three-phase, six-part content analysis process to examine and analyze published guidance on literature searching. Card sorting, Delphi methods, and an online questionnaire were then used to validate our findings and build a code of practice.

**Results:**

Our code of practice for mediated searching lists eighty-five search tasks arranged in performance order, within five progressive levels of search complexity. A glossary of 150 search terms supports the code of practice.

**Discussion:**

The research literature on mediated search methods is sparse and fragmented, lacking currency and a shared vocabulary. A code of practice for mediated searching will provide clarity in terminology, approach, and methods. This code of practice will provide a unified and convenient reference for training a new hire, upholding standards of search service delivery, or educating the next wave of health library professionals.

## Introduction

Although libraries have provided online-mediated search services for more than forty years [1], there is not yet a practice standard to guide execution of searches, train searchers, or evaluate search performance. “Mediated search services” describe the search services offered by libraries and professional librarians, encompassing professional search services, expert search services and related search services.

Four Canadian health services librarians with a workplace need for consistent approaches to mediated searching came together as a working group in June 2014 to address the deficit of a practice standard. Our working group expanded by late 2015 to include eight members from six provinces, four library settings (academia, health services, government, and private sector) and a Master of Library and Information Studies (MLIS) teaching program. Together, members span six search roles and a collective one hundred and twenty-seven years of mediated search experience.

This paper explains the process that led to the creation of a code of practice (Online Supplement, Appendix A) for mediated searching and its associated search glossary (Online Supplement, Appendix B) of frequently used terms in mediated search practice. After four years of reading and research around a “search standard”, the working group’s search for professional guidance moved beyond the library and information sciences (LIS) field and the “practice standard” label. The working group examined standards, guidelines, standards of practice (SOP), and codes of practice. The label “code of practice” was then identified as appropriate for a document integrating both research and professional expertise in mediated search services. A code of practice for mediated search services will help provide assurance for our clientele that our profession’s approach to supporting their work uses the same standard of care and best practices that they must apply in their own practice. It will provide a basis for quality indicators and performance management and help service managers demonstrate the value of library services and of mediated searching within the health care system. The code of practice will provide a foundation for literature search instruction within library schools. It may lead to the development of other best practices or standards for librarianship.

This paper provides an overview of the literature on mediated searching, and details of the process used by the working group to build the components of a code of practice. Applying the Code within a library service and providing an analysis of its use in practice is an area for future research.

### Terminology Used to Organize the Code

- We use the label “steps” for search methods or tasks carried out in the process of mediated searching.
- We use the label “stages” to group the “steps” together throughout the mediated search process (i.e. client engagement, scoping search, etc.).
- We use the label “levels” to identify the increasing complexity and difficulty of mediated searches. Searches at a lower “level” require fewer search “steps”.

### An Overview of the Literature on Mediated Searching

There is a body of literature within health and medicine on searching to support systematic reviews. In comparison, there is very little guidance published for mediated literature searching generally.

Some of the earliest works from the mid-1970s and early 1980s were from the Library and Information Sciences (LIS) literature [2-6], but there were also more recent publications in the health and medicine literature [7-14], including some by health sciences librarians publishing outside the LIS field [15-20]. Of these, a 2018 publication by Cooper et al. [21] examined nine guidance documents, labelling an eight-stage process of searching for systematic reviews “the conventional approach.”

Works by Markey were among the earliest content we found, whereas publications by Booth et al. and Rethlefsen et al. were among the most relevant. Markey’s work spanned almost 35 years [2, 22, 23]. Booth et al.’s “Systematic Approaches to a Successful Literature Review” [15] provided one of the more focused sources of data when it came to gathering information for our code of practice, with essential steps to the search process outlined. Rethlefsen’s 2014 JAMA article [20] outlined a “proposed approach to enhance the quality of review articles through collaboration with medical librarians” and served as a starting point for comparison with earlier [3, 24] and later [21] search process frameworks.

Publications also exist that consider search complexity or suggest that searches should be “fit for purpose”, and that not all searches require the same degree of rigour [26-34]. The literature that we reviewed was mostly comprised of brief outlines of how to conduct mediated searching, and almost always framed this information in the context of teaching other medical or university professionals how to search. The tasks and processes associated with searching are very scattered within the published literature. Library professionals have endeavoured to record their professional searching expertise in pearls dispersed among textbooks, articles, and websites. Information identifying and explaining individual search tasks is also available on database vendor help pages. Publications sometimes described search tasks as operations, tactics, or strategies [14, 35-41]. Some articles modelled search processes [40, 42] or assessed search expertise [43-46]. While these areas were of interest, they were outside the scope of our project.

LIS publications on searching tended to be practice-based after the mid-1990s. These publications did not reference earlier research work and had inconsistencies in terminology, which were at least partly due to the lack of a shared search language. Discussion and identification of search methods appeared in epidemiology, nursing, pharmacy, and other health fields, without reference to early LIS search work.

We found definitions for “expert searcher,” “highly experienced searcher,” and “professional searcher,” [42] but we did not find what we wanted most — that one, landmark publication that integrated and updated 1970s search research. There was no one publication that included most search methods, provided guidance on how to approach different search questions, documented search methods and results, and measured mediated search quality performance to support the concept of expert searcher.

## Methods

We conducted an environmental scan to determine if any similar projects were in progress before project work began in September 2014. We posted to fifteen library and health listservs asking for any information on existing mediated search standards (none were found) or current research or practice-based projects involving mediated search standards. We discovered in-progress, search-related projects; however, they did not have completed publications or outcomes measures that were applicable to our research.

**Fig. 1:**
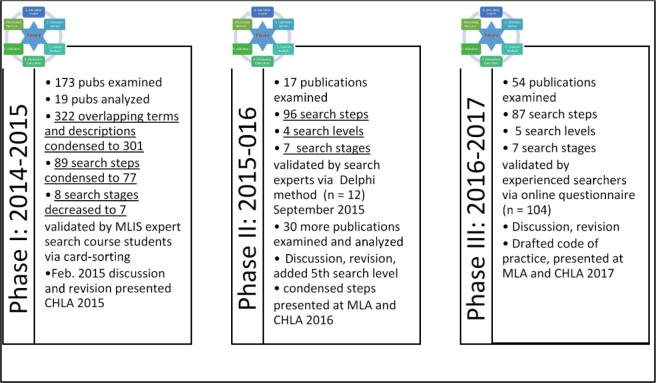
Summaries of study progress following initial work completed in December 2014.

Following this initial work, our project unfolded in three phases over four years (Figure 1).

**Fig. 2:**
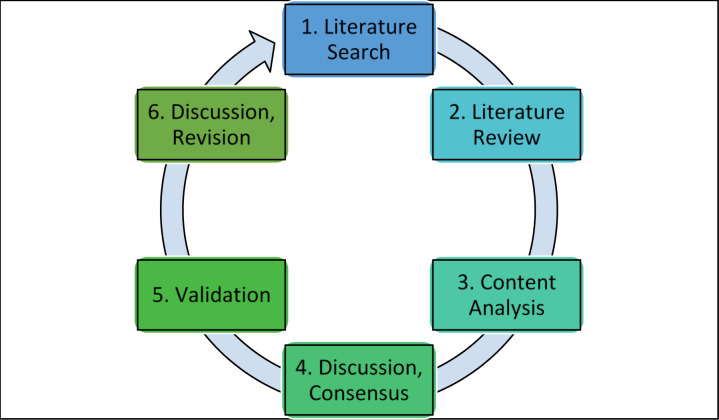
Each phase generally followed this six-part study process.

Each phase generally followed a six-part cyclic process: 1. Literature search, 2. Literature review, 3. Content analysis, 4. Discussion and consensus, 5. Validation, 6. Discussion and revision (Figure 2). Meetings were held virtually using audioconferencing.

**Fig. 3:**
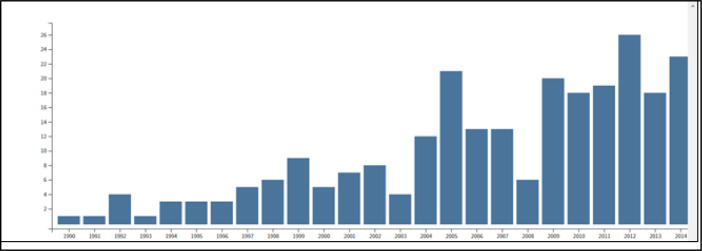
Relative number of publications on mediated searching by year, 1990-2014. From Phase I *Web of Science* Search.

**Fig. 4:**
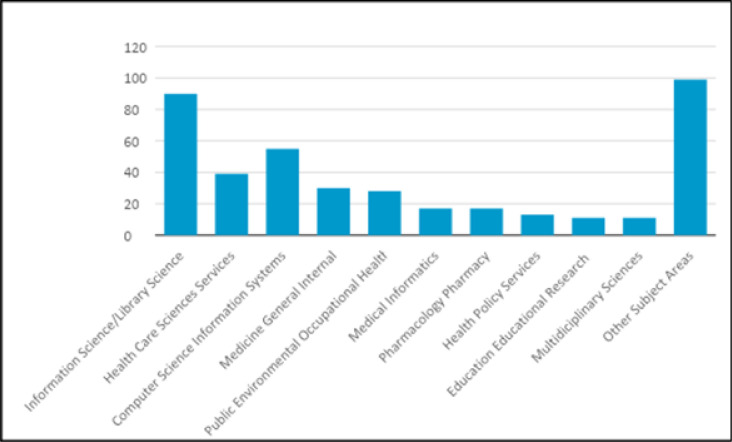
Subject areas of 75% of references retrieved 1990-2014. From Phase I *Web of Science* Search.

Phase I began with a literature review, which was a simple Web of Science scoping search designed to gain an impression of the published literature on mediated search practices — its size, publication year span, and subject areas [TOPIC: ((literature or information) near/2 search*) AND TOPIC: ((librarian OR professional OR expert OR mediat*) near/2 search*]. Results suggested an increasing annual number of potentially relevant publications (Figure 3) with seventy-five per cent of references retrieved in ten Web of Science subject areas (Figure 4).

From these, group members identified the following databases to be searched: *ProQuest ABI/Inform; ACM Digital Library; ProQuest Dissertations & Theses; PubMed MEDLINE; Ovid Embase; EBSCO CINAHL; EBSCO PsycINFO; EBSCO Library Literature & Information Science Index; and EBSCO Library, Information Science and Technology Abstracts (LISTA)*.

Individual working group members searched these databases in the fall of 2014, with update searches in 2015 and 2017. Sensitive searches were designed to identify every possible relevant reference in LIS and biomedical databases, and precise searches were designed to pull only relevant references within business, computer sciences, and management sciences databases. Initially, the group used database limits to restrict results to research-based articles; then, finding retrieval sparse, removed this restriction to include practice-based publications.

We also searched the grey literature, in particular through association web sites for professional LIS, medical, and health association standards that might include directions for searching the literature.

We used citation chaining [47] in *Web of Science* to monitor relevant articles to help identify additional key research articles. We chose RefWorks to manage the 274 references identified for review.

Although our searching was iterative over the four years of our project, and we continued to monitor the literature while writing this article, thus identifying one additional 2018 publication [21], ours was not an exhaustive search, as required for a systematic review simply because we did not identify a cumulative, progressive body of research on either searching or mediated searching.

We found a variety of terms used to describe the search process. With no standard lexicon available, we began the creation of a comprehensive search glossary whereby search types, methods, and related terms are defined and sourced. Glossary development was integral to our work, with terms added and consolidated throughout our project.

**Fig. 5:**
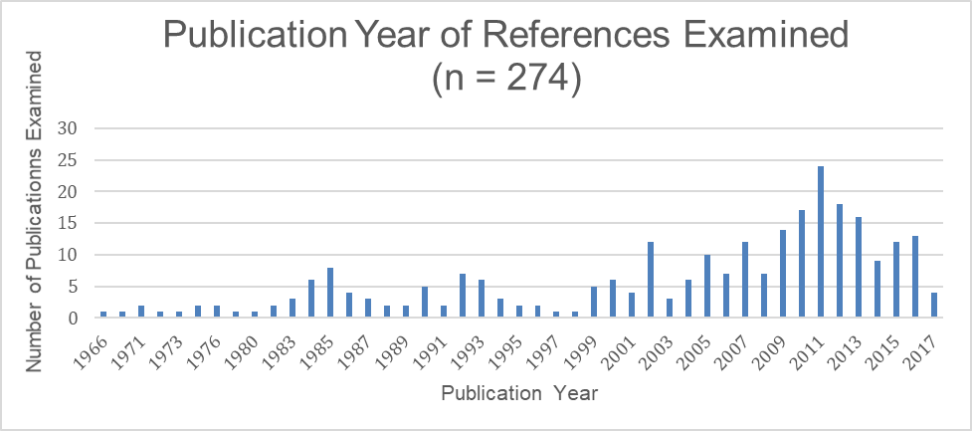
The literature on searching, including mediated searching.

We examined 274 publications dating between 1966 and 2018 (Figure 5) looking for search methods or any guidance on how to conduct a literature search.

Content was analyzed for instructions or descriptions of tasks related to completing a literature search. From this analysis, we built a matrix of mediated search tasks recorded in an Excel spreadsheet.

Table 1 shows how we compared the mechanics of searching as discussed in publications where steps or stages were identified. Gaps in the table indicate a step not addressed in the publication.

**Table 1:** Four of the search frameworks included in content analysis.

1966	1991	2014	2018
Parts of a Search (24)	Seven Stages of an Online Search (3)	Approach to Searching for Review Articles (20)	Eight Key Stages for Searching to Support Systematic Reviews (21) ***Note: the 1st stage, “Who should search”, out of scope for this mediated search study*.**
	1. Reference interview		2. Aims and purpose of literature searching
	2. Tactical overview	1. Define search question(s) and prospective inclusion and exclusion criteria	3. Preparation
		2. Conduct preliminary search to further clarify its scope	
Question			
	3. Database selection	3. Choose data sources based on:	
		a. Database content	
		b. Database platform (vendor, interface)	
		c. Search terms	
		d. The need for non-indexed (grey) literature	
	4. Search strategy formulation	4. Reduce the research question into major concepts	4. The search strategy
		5. Develop search terms and synonyms (controlled vocabulary and text words)	
Interrogation of a source	5. The online search	6. Execute search	5. Searching databases
			6. Supplementary searching
Decision	6. Feedback or reviewing results		
Significant result of aninterrogation			
**!!!!!**“dead end” of a search path			
	7. Presenting final search results		7. Managing references
		8. Document the search procedure	8. Reporting the search process
		9. Document the search results	

As we organized the search tips and directives into categories, different stages in the search process became apparent, and steps within each of these stages emerged. This process helped distill the large list of tasks into generic search steps and to sort them into sequential order.

Over the course of our project, our group reviewed, analyzed, and achieved consensus on our work. We checked our results at the end of each phase as a means of determining the accuracy and completeness of our work.

Our project was initially a quality improvement project, so we began by grouping search tasks within the four-stage Plan-Do-Study-Act Deming Cycle [48]. By the end of phase I, we had extracted 91 search tasks from the literature organized within eight broad stages of the search process, initially labelled as 1. Engage, 2. Plan, 3. Do, 4. Check, 5. Study/Check, 6. Act, 7. Evaluate, and 8. Report.

We used a closed-card sorting exercise to determine whether and how our sorting and grouping was similar to or different from that of three other similarly-sized groups. Card-sorting exercises use pictures, cards, or other objects to determine how people categorize or prioritize issues and see relationships between them, such as what does or does not belong [49]. In closed-card sorting, participants sort cards into predefined groups [50]. We separated MLIS students enrolled in an expert search course (n=18) into three groups. Each group participated in the study by sorting 91 search task cards and 8 numbered search stage cards. We then asked them to first sort the cards by stage, and then organize the task cards within each stage.

Analysis of this validation entailed identifying points where cards were sorted or ordered differently than ours, then considering each deviation. We also reworded tasks that required clarification during this process.

Overlap in cards sorted between two consecutive sections, “Check” and “Study/Check” suggested there might not be enough of a difference between these to separate them. We decided to merge them, reducing the number of stages from eight to seven.

Phase II of our work was an in-depth review of the search steps, their sequence and placement within our proposed search stages, and the clarity and accuracy of wording in the labels we established to describe them. We also identified four levels of search complexity, from a search for a single fact, to a search for a systematic review.

We used the RAND Corporation’s Delphi method, recognized for its effectiveness in gathering information from individuals on their area of expertise [51, 52], to ask subject experts to review and comment on our work and to point out any steps, stages, or literature that might be overlooked. We approached the twelve published researchers identified from our literature review to comment on our work by answering two questions:

1. Have you come across an authoritative taxonomy of search types? If yes, what is it?
2. If not, what do you think of the following labels and definitions? Please point out anything that you feel is missing.

We also sent these questions to MEDLIB-L, CANMEDLIB, and the Expertsearching listservs, receiving a total of 104 responses. These responses were compiled with those from the twelve researchers.

Analysis took the form of working through each response as a group, to consider each comment and decide whether we shared each view and whether an addition or revision was warranted. After working through our experts’ comments and reviewing their 30 suggested publications that were new to us, we added one more level of search complexity. This was inserted as level 3, representing the type of search most frequently completed by mediated searchers.

Phase III began in late 2016 with a literature search, review, and analysis of work completed in the other phases of the project. Phase III was concluded in 2017, with a Regina Qu’Appelle Health Region Research Ethics Board-approved online questionnaire (Online Supplement, Appendix A) to validate our work. We used Dalhousie University’s Opinio Survey Service (http://www.objectplanet.com/opinio/.)

To create the survey, working group members provided 14 search requests and classified them within our five levels of search complexity. Search researchers, authors publishing on search topics, and mediated searchers were asked to choose one of these 14 exemplary searches and describe how they handled a recent similar search. Respondents assessed the level of difficulty (“search level”) and then worked their way through the steps and stages of the search process, indicating which steps they had carried out in their own work. The purpose of this validation study was to highlight areas where the published guidance on searching identified an approach that deviated from what experienced mediated searchers do in practice.

Of 104 questionnaire respondents, most were self-selected (n = 91) in response to invitations sent on April 3, 2017 to the Expertsearching listserv, with the target population of 855 members. Convenience sampling (n = 13) was used to increase Canadian responses after the questionnaire closed. Working group members reviewed respondents' locations, identified under-represented provinces, reopened the questionnaire, and then asked search professionals in these areas to complete the forms.

**Fig. 6:**
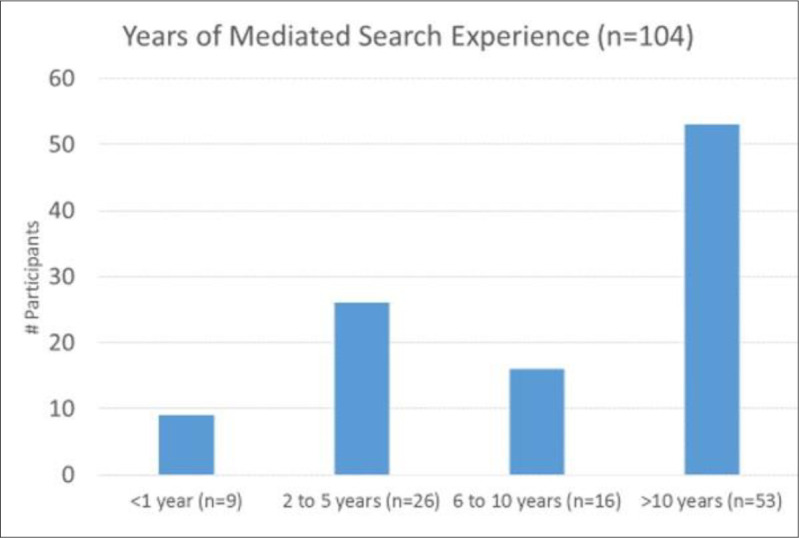
Years of mediated search experience (n = 104). From Phase III validation questionnaire respondents.

**Fig. 7:**
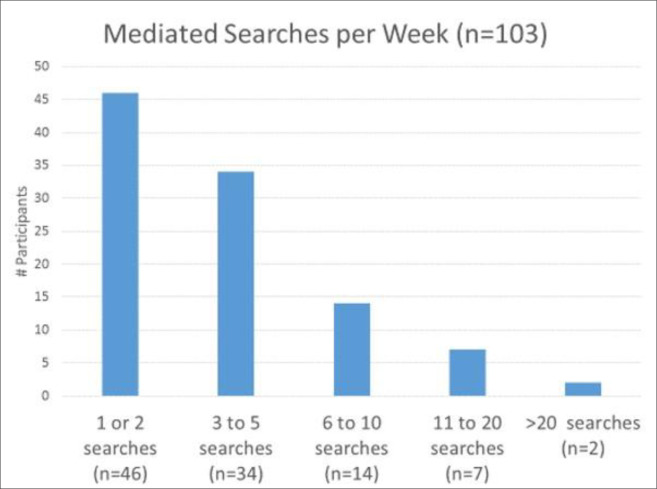
Mediated searches executed weekly (n = 103). From Phase III validation questionnaire respondents.

**Fig. 8:**
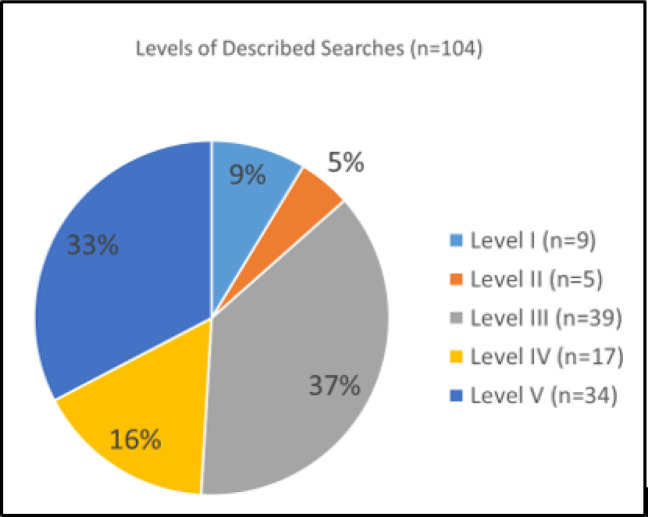
Level of searches described to answer questions about search methods (n = 104). From Phase III validation questionnaire respondents.

We gathered data on each respondent’s age, country, type of library, education, years as a mediated searcher, and average number of searches per week, as well as the exemplary search chosen, the client group, and the search purpose or information needed. Respondents with more years of mediated search experience did not always carry out higher numbers of searches per week or chose to describe higher level searches. We analyzed overlapping subgroups identified through respondents’ years of mediated search experience (Figure 6), their average number of searches per week (Figure 7), and the complexity of the exemplary search they selected (Figure 8).

We imported data from the Opinio Web survey system to an Excel spreadsheet for data analysis. We wanted to see where respondents agreed with each other about how to search, and where they agreed with published guidance on searching, so we analyzed responses in subgroups according to the level of searches they described. We arranged the search steps in sequence, and analyzed all responses by subgroup according to the level of search they described. We then arranged levels of searches in bands of rows by “level of search” — one row per step for each search described.

To get a high-level impression of within-group agreement, we used simple counts to calculate a value for x/y = c. X = number that carried out a step, y = number in the subgroup, and c = a calculated value to show congruency within the subgroup. For example: If among the subgroup describing level I searches (n = 9), all 9 indicated that they carried out a step, our calculation was 9/9 = 1; if none carried out a step, our calculation was 0/9 = 0. We considered “1” and “0” to be complete agreement. If some carried out a step and others did not, the split in responses indicated less agreement. Responses close to 0.50, + 0.09 — for example, 4/9 = 0.45 or 5/9 = 0.55 — were considered to disagree. We applied this formula for all steps within each search level, and used a search-and-replace command to colour cell backgrounds by range. Green cells indicate a shared approach; red cells indicate variations in approaches. Using these values, we applied Excel Find and Replace to fill cell backgrounds of values, by range, with colours shown in Table 2.

**Table 2:** Cell background colours used to highlight congruency between what expert searchers said they did and the published guidance literature on searching.

Congruency range		Respondent Subgroups	Published literature suggests Step is required;c/1=Cell Fill Colour	Published literature suggests Step not required;c/-1=Cell Fill Colour
0.85	1.00	All or almost all carried out the step	Very High Agreement	Disagreement
0.75	0.84	Most carried out the step	High Agreement	Disagreement
0.60	0.74	Slightly more than half carried out the step	Moderate Agreement	Disagreement
0.50	0.59	Just over half carried out the step and just under half did not	Low agreement	Very Low Agreement
0.40	0.49	Just under half carried out the step and just over half did not	Very Low Agreement	Low agreement
0.30	0.39	Around 2/3 did not carry out the step	Disagreement	Moderate Agreement
0.25	0.16	Most did not carry out the step	Disagreement	High Agreement
0.15	0.00	All or almost all did not carry out the step	Disagreement	Very High Agreement

**Fig. 9:**
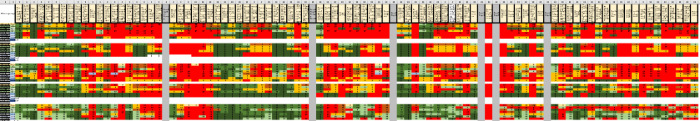
A high-level view of validation questionnaire respondents’ (n = 104) agreement on searching.

The resulting grid was almost all red-filled cells, which gave us the impression of considerable disagreement within subgroups by search level (Figure 9). Online Supplement, Appendix B allows a closer look at a similar spreadsheet.

We then opted to include in our analysis only responses from more experienced searchers, those with six or more years of mediated search experience (n = 69) or who typically executed six or more mediated searches (n = 23) per week. We arranged the search steps in sequence in row one, with cells as column headers. We arranged levels of searches in bands of rows, with each row a subgroup of a more experienced searcher. Consequently, each step consumed 25 rows — one for each of five subgroups of searchers per search level for five levels. Results of this analysis resulted in more green-filled cells in the grid, indicating more agreement within subgroups with more search experience.

To see where respondents agreed with published guidance on searching regarding steps required for a particular level, we conducted a second analysis using a simple calculation. We calculated a value for c/1 for those steps we saw as required for a level, and c/‒1 for those steps we saw as not required for a search level.

**Fig. 10:**
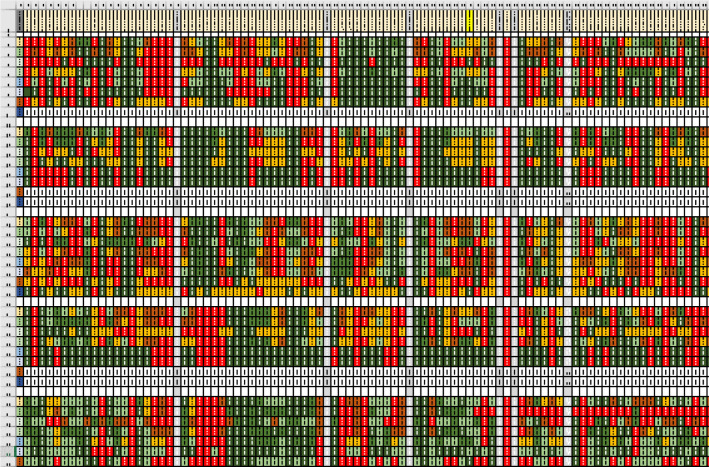
A high-level view of the degree to which seven subgroups of more experienced respondents “agreed” with the search guidance literature.

We arranged search steps in sequence in row one, with cells as column headers. We arranged levels of searches in bands of rows, with each row containing a subgroup of a more experienced searcher. Consequently, each step for a particular search level consumed 25 rows, creating strips and patches of congruency (green cells) and lack of congruency (red cells), as shown in Figure 10. Green cells indicate where most subgroup respondents described carrying out a step identified in the published guidance literature. Red cells indicate where subgroup participants did not describe carrying out a step we identified in the published guidance literature.

As the next step in this analysis, we used the same approach to look at more experienced searchers as a single group, producing a smaller, more condensed grid. Working group members followed up with 12 clusters of red cells. Members presented each search step within their assigned clusters back to the group with recommendations for revision, no change, or further discussion. After several minor phase III revisions, we finalized our code of practice.

## Results

In May of 2018, we created our proposed code of practice (included in full in the supplementary material for this article). The code identifies 85 unique search steps listed across five search types (called levels) and characterized by increasing rigour and attention to detail. The amount and type of recommended steps vary depending on the level of the search. We grouped the steps into seven search stages from the initial client engagement stage to the final reporting stage.

## Search Stages with Numbers of Steps

Search stage 1 — ***Client Engagement*** (20 Steps): Capturing the client’s information need

Search stage 2 — ***Initial Planning*** (18 Steps): Studying how to best meet the client’s information need by outlining or sketching out one or more “fit for purpose” approaches

Search stage 3 — ***Scoping Search*** (10 Steps): Testing one or more approaches to establish the best course of action

Search stage 4 — ***Resource-Specific Search Planning*** (11 Steps): Selecting and planning the best approach for each source to be searched

Search stage 5 — ***The Search*** (1 Step): Executing the search in each resource selected

Search stage 6 — ***Evaluation*** (5 Steps): Evaluating how well the search worked in each resource

Search stage 7 — ***Reporting*** (20 Steps): Recording the completed work to meet the needs for service record-keeping and client information needs and uses

## Search Levels with Descriptions

***Search Level I:***

To bridge an information gap with a needed fact

***Search Level II:***

To increase an individual's own understanding of an issue

***Search Level III:***

To find information to apply immediately for an individual patient’s care
To help inform the creation of patient education
To gather content for education such as lectures, workshops, presentations
  To support a decision to purchase a product or resource
  To inform a student assignment such as a term-paper or an essay
  To write an internal report or other internal document or policy
  For non-clinical organizational planning

***Search Level IV:***

To inform knowledge synthesis
To inform research proposals

***Search Level V:***

To inform research
To develop clinical guidelines
To support a systematic review, meta-analysis or health technology assessment, etc.

We conducted a questionnaire response analysis to determine congruency between respondents and our model. It showed inconsistencies in approaches to searching generally, between respondents and our research group, within and between subgroups. Differences in descriptions of how to carry out similar searches within subgroups of searchers who received MLS degrees within several years of each other may indicate inconsistencies in search education and training. Some questionnaire respondents indicated their uncertainty with the search terminology used in certain steps. A lack of certainty with terminology further confirmed the need for a glossary of search terms, which we developed in tandem with our code of practice.

## Discussion & Conclusions

The current version of our glossary contains more than 150 terms related to searching for health information. Each glossary term definition contains relevant sources by way of citation (and a link where applicable). We used the most current definition unless an earlier definition provided a better illustration. The glossary is a living document, which will grow and undergo updates, as needed. It is our hope that this glossary will create a standard set of definitions and facilitate coherent conversations among search professionals.

While we did find publications that grouped tasks as a framework or series of stages to consider when performing a search, there was a general lack of congruency among these. Our intent was to research and document what was described in the literature, and then apply our own examples and experience to the information uncovered. In this manner, we intended to create a usable, accessible standard that encompasses the variety of literature searches done in medical libraries without being too complex. While there is never really one truly “correct” way to search, we hope having best practice laid out in such a way will be useful for maintaining the search standards of library professionals. We grappled with how our code of practice should encompass all the helpful details of existing frameworks into a usable document. We did determine that since the PRESS Peer Review of Electronic Search Strategies [17] already exists, we would not need to include a new peer review process for searches.

As we sorted the steps of the search process into the chronological stages of searching, it became clear that not all steps are required for all types of searches. We found the need to define the various levels of complexity that are possible in a professional search service. While examining the literature on searching from our review, we found four types of search questions identified: reference (factual search), “quick education” search, semi-structured search, and systematic search. Adding the concept of search types and their associated levels of complexity helped further organize our code of practice document. Some of our search steps are required for a search of any level of complexity, while others are only recommended for searches with a greater demand for detail and thoroughness. Adding levels allowed us to keep both the very simple and more complicated steps in one unified code of practice document. Providing room for these choices within the code of practice speaks to the benefit of being flexible and scalable in our search techniques, able to adapt and add increasingly complex techniques, as needed. Naming the search levels was a challenge because terms such as “semi-structured search” mean many things to a wide variety of mediated search services professionals. In the end, we landed on labelling search levels using numbers, and adding descriptive definitions for each numbered level.

The online questionnaire used as our final validation highlighted inconsistencies in approaches used by mediated searchers. A code of practice will provide clarity in terminology, approach and methods. It will align with the practice standards and guidelines developed by health care professional organizations and maintain the relevance and value of health services libraries within the health care system. Standards enable us to provide a consistent service experience to our users, especially within multi-site services with both physical library and virtual environment settings.

## Study Limitations

We designed our project to draw on published LIS research related to mediated searching and then found that body of literature both sparse and fragmented, and with perhaps a greater reliance on self-evaluation studies than empirical research.

We designed our validation questionnaire to highlight areas of our work needing review. Although this part of our study was validation, not empirical research, we believe data collected does highlight important areas for further work.

Having a dedicated researcher would have expedited our project, provided greater focus, and allowed us to complete our study in a timelier manner.

## Further Research and Development

Writing Mediated Searching: A Code of practice is simply the first step in creating guidance documents for the profession of health sciences and medical librarianship. It is our hope that continued investment in the *Code* is undertaken to better identify issues that may be missing or include future practice as it evolves. In order to provide a platform for further research and development, and allow for ongoing access to the code of practice, it would be beneficial for national organizations such as the Canadian Health Libraries Association and the Medical Library Association to sponsor further research. As a first step in applying the Code in practice, we hope that libraries will consider classifying their searches using the Levels of searches in order to be more consistent in recording the types of literature searches performed across our institutions. There are many opportunities for future library services researchers to develop this code of practice further. Adding qualifiers (“may,” “should,” or “must”) for each search step, exploring the nature of mediated search expertise from novice to expert, looking at the average length of time to complete searches (by level), or expanding on aspects of search quality and performance monitoring are some of these opportunities.

## Supplementary Material




